# Kidney Response to Chemotherapy-Induced Heart Failure: mRNA Analysis in Normotensive and *Ren-2* Transgenic Hypertensive Rats

**DOI:** 10.3390/ijms22168475

**Published:** 2021-08-06

**Authors:** Šárka Jíchová, Olga Gawryś, Elżbieta Kompanowska-Jezierska, Janusz Sadowski, Vojtěch Melenovský, Lenka Hošková, Luděk Červenka, Petr Kala, Josef Veselka, Věra Čertíková Chábová

**Affiliations:** 1 Center for Experimental Medicine, Institute for Clinical and Experimental Medicine, 14021 Prague, Czech Republic; savr@ikem.cz (Š.J.); ludek.cervenka@ikem.cz (L.Č.); kaap@ikem.cz (P.K.); 2 Department of Renal and Body Fluid Physiology, Mossakowski Medical Research Institute, Polish Academy of Sciences, PL02-106 Warsaw, Poland; gawo@ikem.cz (O.G.); ekompanowska@imdik.pan.pl (E.K.-J.); jsadowski@imdik.pan.pl (J.S.); 3 Department of Cardiology, Institute for Clinical and Experimental Medicine, 14021 Prague, Czech Republic; vome@ikem.cz (V.M.); lenka.hoskova@ikem.cz (L.H.); 4 Department of Pathophysiology, 2nd Faculty of Medicine, Charles University, 15006 Prague, Czech Republic; 5 Department of Cardiology, University Hospital Motol and 2nd Faculty of Medicine, Charles University, 15006 Prague, Czech Republic; josef.veselka@fnmotol.cz; 6 Department of Nephrology, 1st Faculty of Medicine, Charles University, 12108 Prague, Czech Republic

**Keywords:** chemotherapy-induced heart failure, doxorubicin, hypertension, kidney, renin-angiotensin-aldosterone system, endothelin system, cytochrome P-450, renal adrenergic system

## Abstract

The aim of the present study was to perform kidney messenger ribonucleic acid (mRNA) analysis in normotensive, Hannover Sprague–Dawley (HanSD) rats and hypertensive, *Ren-2* renin transgenic rats (TGR) after doxorubicin-induced heart failure (HF) with specific focus on genes that are implicated in the pathophysiology of HF-associated cardiorenal syndrome. We found that in both strains renin and angiotensin-converting enzyme mRNA expressions were upregulated indicating that the vasoconstrictor axis of the renin–angiotensin system was activated. We found that pre-proendothelin-1, endothelin-converting enzyme type 1 and endothelin type A receptor mRNA expressions were upregulated in HanSD rats, but not in TGR, suggesting the activation of endothelin system in HanSD rats, but not in TGR. We found that mRNA expression of cytochrome P-450 subfamily 2C23 was downregulated in TGR and not in HanSD rats, suggesting the deficiency in the intrarenal cytochrome P450-dependent pathway of arachidonic acid metabolism in TGR. These results should be the basis for future studies evaluating the pathophysiology of cardiorenal syndrome secondary to chemotherapy-induced HF in order to potentially develop new therapeutic approaches.

## 1. Introduction

Heart failure (HF) has become a major public health problem, particularly in developed countries, affecting currently more than 6.5 million people in the United States of America and 9.2 million people in the European Union. The yearly increase in the number of new patients is estimated at 1.1 million [1,2]. The increase in the prevalence of HF is attributed, at least in part, to the improvement in the treatment of acute coronary syndromes and also of non-ischemic cardiovascular diseases. Remarkably, the progress in the treatment (e.g., early coronary reperfusion by primary percutaneous intervention) has decreased the mortality rate but not the morbidity. Somewhat paradoxically, the number of surviving patients who ultimately develop HF has augmented [3].

HF is a clinical syndrome showing progressive aggravation, despite recent pharmacological advances. The prognosis of the patients remains poor, particularly when HF is accompanied by kidney dysfunction (“cardiorenal syndrome”) [1,4,5,6,7,8]. Therefore, there is an urgent need for new treatment strategies, which require a better understanding of the pathophysiological mechanism(s) underlying the progression of HF. This can be achieved using small animal models, despite some apparent limitations [9,10]. Over the last 40 years, the models were applied to study both ischemic injury-induced HF [myocardial infarction (MI) induced with coronary artery ligation] and non-ischemic injury-induced HF models [chronic pressure overload-induced by transverse aortic constriction or chronic volume overload-induced by aorto-caval fistula (ACF)]. All these models were comprehensively characterized [9,10,11,12,13,14,15] and brought pioneering achievements. For instance, the application of the MI model first enabled the demonstration that angiotensin-converting enzyme inhibitors (ACEi) attenuate HF and improve the long-term survival rate after the infarction [14,15]. This has been confirmed in clinical studies [16,17], and ACEi has been established as the cornerstone therapy for HF [1,4,5,6,7].

In contrast, the value of small animal models for the study of chemotherapy-induced HF is only poorly defined. This is so even though cardio-oncology has now emerged as a new clinical and research specialty, bringing novel cancer therapies, which have dramatically improved the prognosis in patients with various cancer diseases. Unfortunately, the improvement is commonly associated with cardiovascular side effects [18,19,20,21].

Doxorubicin (DOX), one of the anthracycline drugs, is a standard anticancer agent showing well-documented cardiotoxicity [22,23,24,25]. Antracyclines’ side effects include impairment of left ventricular (LV) ejection fraction [26] and may lead to cardiorenal syndrome, a life-threatening complication of cancer survivors that requires the development of new treatment strategies. Small animal models were dominantly employed to study the mechanism(s) underlying acute DOX-induced cardiotoxicity and to develop protective measures against DOX-induced cardiotoxicity [27,28]. Long-term effects of DOX on cardiac function, in particular, on the development of HF, were also evaluated, and the results indicate that it is a suitable model of chemotherapy-induced HF [10,29,30], but still, underlying mechanism(s) responsible for the development of chemotherapy-induced HF are poorly understood [10,29,30,31,32]. Considering the growing need to investigate the pathophysiology and to discover novel therapeutic measures for chemotherapy-induced HF, we recently performed an in vivo study that characterized cardiac morphological structure and function parameters in rats with DOX-induced HF. Since hypertension and hyperactivity of the renin-angiotensin-aldosterone system (RAAS) are considered as risk factors for the development of chemotherapy-induced HF [21,22,23,24]), the study was performed in *Ren-2* transgenic rats (TGR), in which endogenous activation of the RAAS and hypertension are combined [33] Moreover, to gain a better insight into the possible role of potential compensatory activation of systemic and intrarenal neurohormonal systems, plasma and kidney concentrations of catecholamines, angiotensin II (ANG II), and angiotensin 1-7 (ANG 1-7) were determined. In this in vivo study, we found that two weeks after cessation of DOX administration [DOX was given in cumulative dose of 15 mg/kg body weight (BW) in six intraperitoneal (*i.p*.) injections over two weeks, which is a standard approach for introducing DOX-induced cardiomyopathy] [27,34], the TGR as well as control HanSD rats were showing signs of “chemotherapy-induced HF with reduced ejection fraction” (HFrEF) but in the former impairment of cardiac systolic function was more pronounced and there were initial signs of compensatory activation of neurohormonal systems [35]. Therefore, we concluded that DOX-induced HF, particularly in TGR, is an optimal model for studying pathophysiological aspects of chemotherapy-induced HFrEF.

Nevertheless, a limitation of our recent study was the lack of attempt to identify potential biomarkers and molecular indices, which could help develop a novel therapeutic approach in this form of HFrEF, particularly with the focus on the markers for HF-associated cardiorenal syndrome. Therefore, in the present study, we performed kidney messenger ribonucleic acid (mRNA) expression analysis in TGR and HanSD rats two weeks after the end of DOX treatment, with a particular focus on the genes that were previously implicated in the pathophysiology of HF-associated cardiorenal syndrome [7,36]. The main aim of the study was to characterize kidney mRNA expression of such selected biomarkers in the initial phase of chemotherapy-induced HFrEF, and compare the results in hypertensive TGR with those in normotensive HanSD rats. In order to confirm that the obtained kidney mRNA expressions represent changes related to HF- associated cardiorenal syndrome, we evaluated the effects of DOX on cardiac weights and on the left ventricle (LV) mRNA expression of biomarkers that are recognized to be changed in HF [37,38,39].

## 2. Results

**Figure 1:**
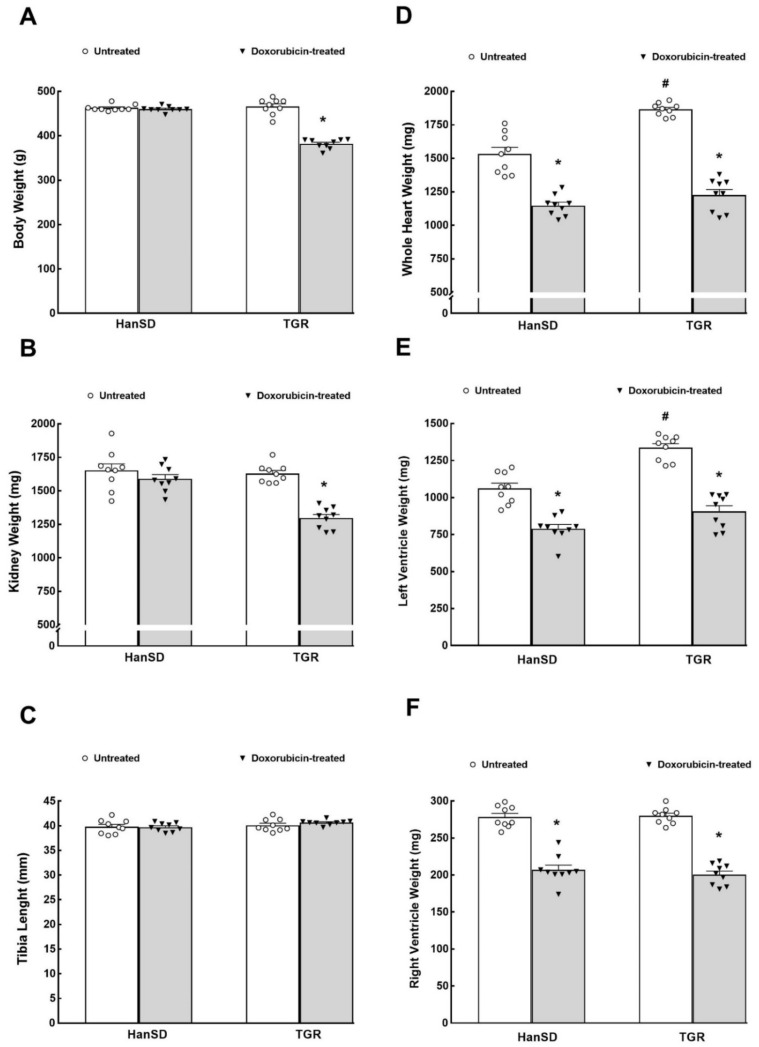
Body and organ weight parameters. Body weight (**A**), kidney weight (**B**), tibia length (**C**), whole heart weight (**D**), left ventricle weight (**E**) and right ventricle weight (**F**) in untreated and doxorubicin-treated normotensive, transgene-negative Hannover Sprague–Dawley (HanSD) and hypertensive, *Ren-2* transgenic (TGR) rats. * *p* < 0.05 compared with untreated animals of the same strain. ^#^ *p* < 0.05 versus HanSD rats within the same protocol. Statistical comparison was made by one-way ANOVA analysis.

As shown in Figure 1A, DOX treatment did not significantly decrease body weight in HanSD rats but did so in TGR. DOX treatment did not cause any change in kidney weight in HanSD rats but decreased it significantly in TGR (Figure 1B). As shown in Figure 1D,E, untreated TGR revealed significantly higher whole heart and LV weights as compared with untreated HanSD rats, but there were no significant differences in right ventricle (RV) weights between them (Figure 1F). DOX treatment caused significant decreases in the whole heart weight, LV and RV weights in HanSD rats, and TGR as compared with weights in counterparts without DOX administration. DOX treatment elicited significantly greater decreases in whole and LV weights in TGR as compared with HanSD rats (−34.3 ± 0.4 vs. −25.1 ± 0.5% and −32.3 ± 0.5 vs. −25.7 ± 0.3%, respectively, *p* < 0.05 in both cases), but caused similar decreases in RV weights. On the other hand, DOX administration did not result in any alteration in tibia length in any group (Figure 1C), indicating that the reduced body weights, kidney, and heart weights are not a consequence of general growth retardation.

**Figure 2:**
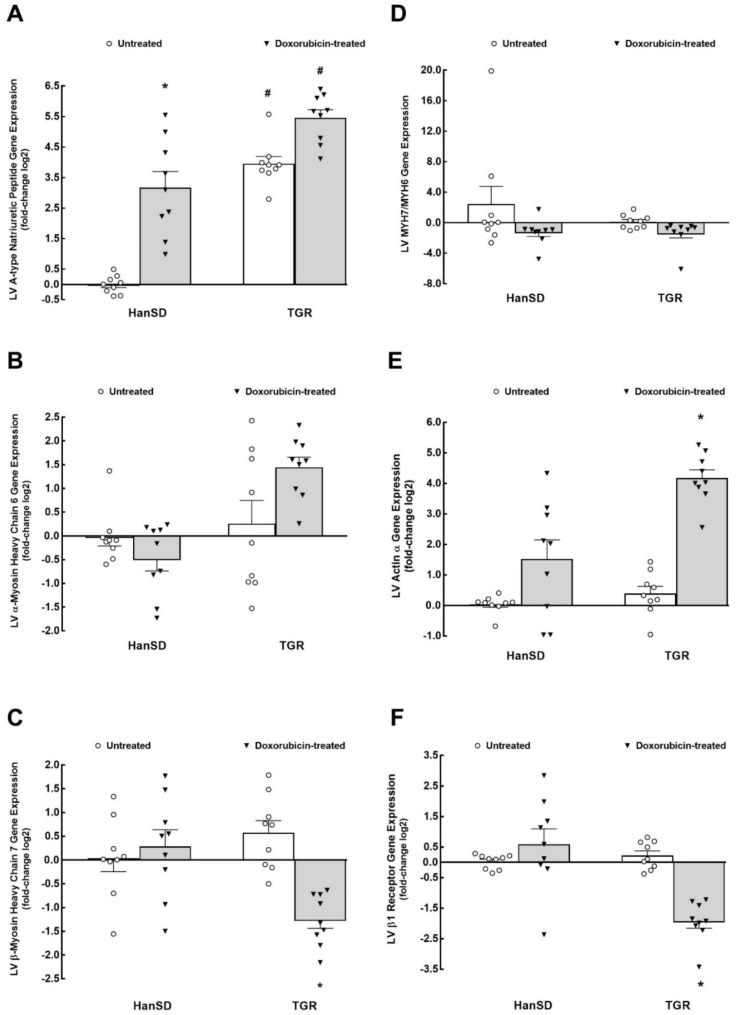
The first part of left ventricle (LV) mRNA expression. LV natriuretic peptide type A (**A**), α-myosin heavy chain isoform 6 (*MYH6*) (**B**), β-myosin heavy chain isoform 7 (*MYH7*) (**C**), ratio *MYH7/MYH6* (**D**), α actin (**E**), β1 adrenergic receptors (**F**) gene expression in untreated and doxorubicin-treated normotensive, transgene-negative Hannover Sprague–Dawley (HanSD) and hypertensive, *Ren-2* transgenic (TGR) rats. * *p* < 0.05 compared with untreated animals of the same strain. ^#^ *p* < 0.05 versus HanSD rats within the same protocol. Statistical comparison was made by one-way ANOVA analysis.

**Figure 3:**
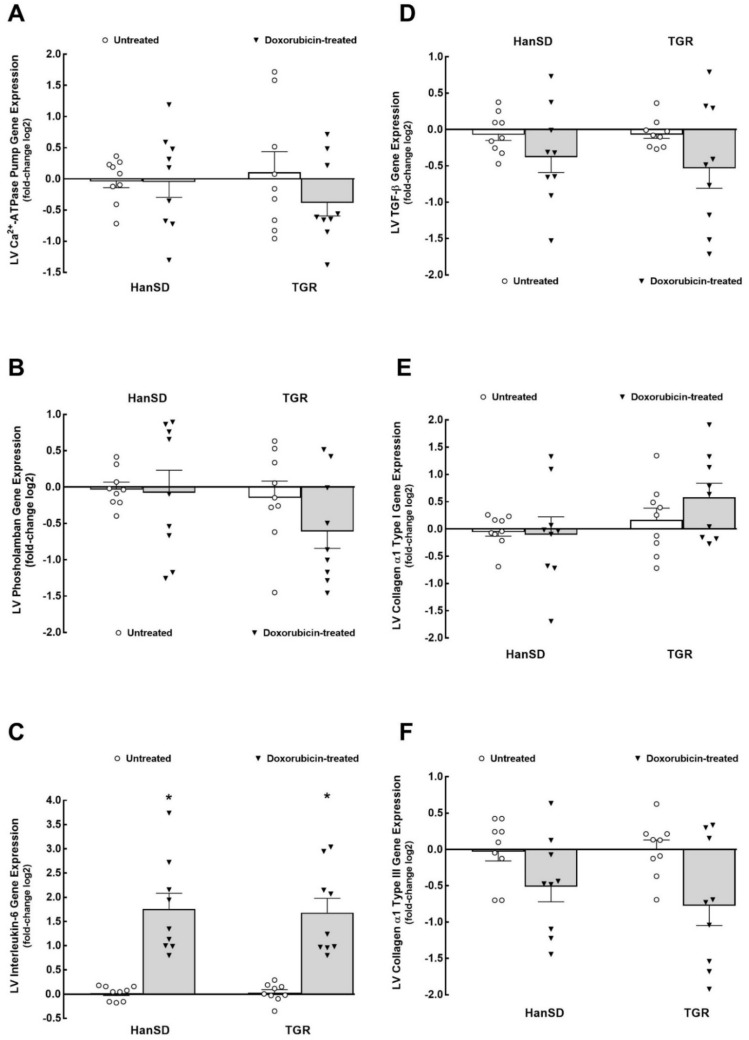
The second part of the left ventricle (LV) mRNA expression. LV Ca^2+^-ATPase pump (**A**), phosholamban (**B**), interleukin-6 (**C**), transforming growth factor beta (*TGF-β*) (**D**), collagen α1 type I (**E**) and collagen α1 type III (**F**) gene expression in untreated and doxorubicin-treated normotensive, transgene-negative Hannover Sprague–Dawley (HanSD) and hypertensive, *Ren-2* transgenic (TGR) rats. * *p* < 0.05 compared with untreated animals of the same strain. Statistical comparison was made by one-way ANOVA analysis.

Figure 2 and Figure 3 summarize the effects of DOX treatment on LV mRNA expression. As shown in Figure 2A, the natriuretic peptide type A (*Nppa*) gene expression in the LV was significantly higher in untreated TGR as compared with untreated HanSD rats. The DOX treatment significantly increased *Nppa* gene expression in HanSD rats as well as TGR, but to a greater extent in the latter, i.e., LV *Nppa* expression was significantly higher in TGR as compared with HanSD rats. There were no significant differences in the LV gene expression of myosin heavy chain α isoform (*MYH6*), myosin heavy β isoform (*MYH7*), and their ratios (*MYH7/MYH6*) in both untreated HanSD rats and TGR and DOX treatment did not alter it in either strain (Figure 2B–D). As shown in Figure 2E, there were no significant differences in the α actin, skeletal muscle mRNA expression in the LV between untreated HanSD rats and untreated TGR and DOX treatment did not alter it in HanSD rats, but caused a significant rise in TGR. There were no significant differences between untreated HanSD rats and untreated TGR in LV mRNA expression of β1 adrenergic receptors, and DOX treatment did not change them in HanSD rats but decreased it in TGR (Figure 2F).

As shown in Figure 3A, there were no significant differences in the ATPase, Ca^2+^, cardiac muscle, slow twitch, type 2 gene expression in the LV (a gene that encodes the sarco/endoplasmatic reticulum Ca^2+^-ATPase—so-called SERCA) between untreated HanSD rats and untreated TGR and DOX treatment did not modify them in either group. Likewise, there were no significant differences in the phospholamban gene expression in the LV between untreated HanSD rats and untreated TGR, and DOX treatment did not alter them (Figure 3B). As shown in Figure 3C, there were no significant differences in the interleukin-6 gene expression in the LV between untreated HanSD rats and untreated TGR, and DOX treatment caused similar significant increases in HanSD rats as well as TGR. There were no significant differences in transforming growth factor beta (*TGF-β*), collagen α1 type I, and collagen α1 type III gene expression in LV between untreated HanSD rats and untreated TGR and DOX treatment did not alter it in either strain (Figure 3D–F).

**Figure 4:**
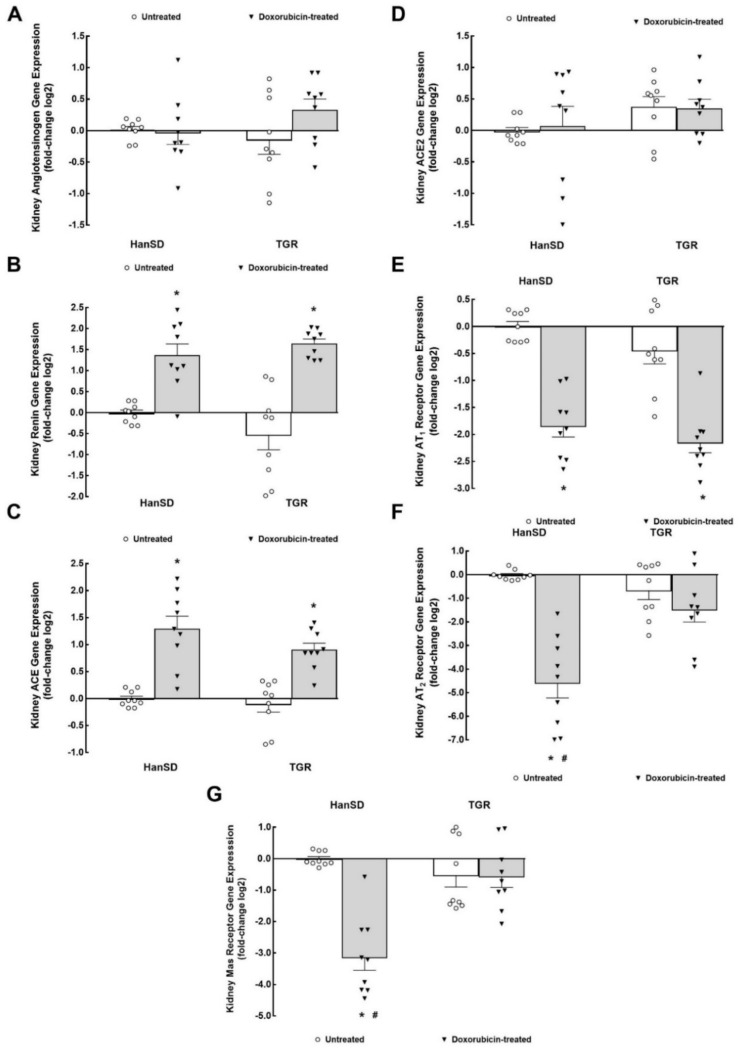
The first part of kidney mRNA expression. Kidney angiotensinogen (**A**), renin (**B**), angiotensin-converting enzyme (*ACE*) (**C**), angiotensin-converting enzyme type 2 (*ACE2*) (**D**), angiotensin II type 1 (*AT*_1_) receptor (**E**), angiotensin II type 2 (*AT*_2_) receptor (**F**) and Mas receptor (**G**) gene expression in untreated and doxorubicin-treated normotensive, transgene-negative Hannover Sprague–Dawley (HanSD) and hypertensive, *Ren-2* transgenic (TGR) rats. * *p* < 0.05 compared with untreated animals of the same strain. ^#^ *p* < 0.05 versus TGR within the same protocol. The values are means ± SEM. Statistical comparison was made by one-way ANOVA analysis.

**Figure 5:**
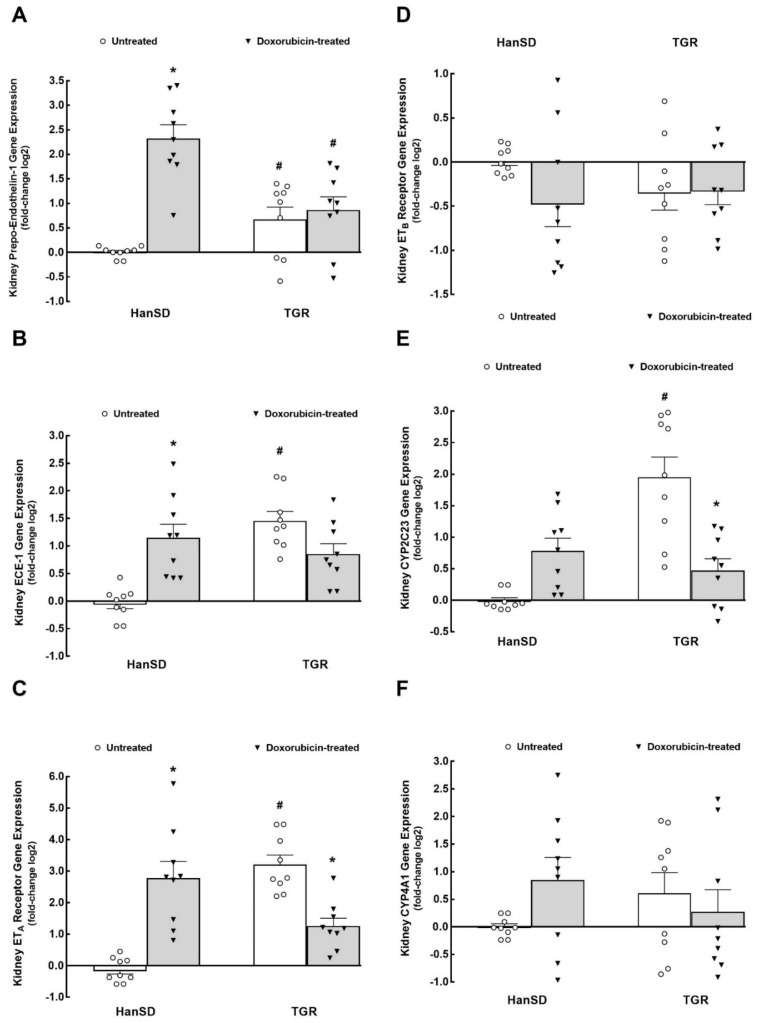
The second part of kidney mRNA expression. Kidney prepro-Endothelin-1 (**A**), endothelin-converting enzyme type 1 (*ECE-1*) (**B**), endothelin type A (*ET_A_*) receptor (**C**), endothelin type B (*ET_B_*) receptor (**D**), cytochrome P-450 subfamily 2C23 (*CYP2C23*) (**E**) and cytochrome P-450 subfamily 4A1 (*CYP4A1*) (**F**) gene expression in untreated and doxorubicin-treated normotensive, transgene-negative Hannover Sprague–Dawley (HanSD) and hypertensive, *Ren-2* transgenic (TGR) rats. * *p* < 0.05 compared with untreated animals of the same strain. ^#^ *p* < 0.05 versus HanSD rats within the same protocol. The values are means ± SEM. Statistical comparison was made by one-way ANOVA analysis.

**Figure 6:**
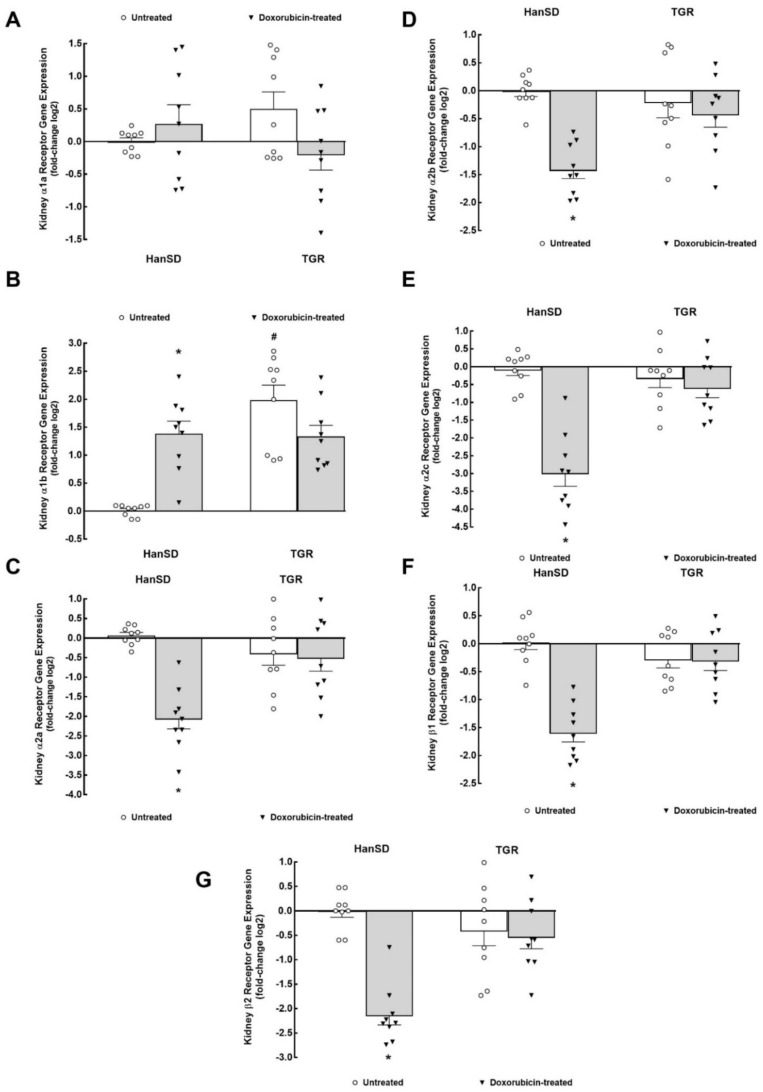
The third part of kidney mRNA expression. Kidney α1 subtype a (α1a) adrenergic receptor (**A**), α1 subtype b (α1b) adrenergic receptor (**B**), α2 subtype a (α2a) adrenergic receptor (**C**), α2 subtype b (α2b) adrenergic receptor (**D**), α2 subtype c (α2c) adrenergic receptor (**E**), β adrenergic receptor type 1 (β1) (**F**) and β adrenergic receptor type 2 (β2) (**G**) gene expression in untreated and doxorubicin-treated normotensive, transgene-negative Hannover Sprague–Dawley (HanSD) and hypertensive, *Ren-2* transgenic (TGR) rats. * *p* < 0.05 compared with untreated animals of the same strain. ^#^ *p* < 0.05 versus HanSD rats within the same protocol. The values are means ± SEM. Statistical comparison was made by one-way ANOVA analysis.

Figure 4, Figure 5 and Figure 6 summarize the effects of DOX treatment on kidney mRNA expression. As shown in Figure 4A, they were no significant differences between untreated HanSD rats and untreated TGR in angiotensinogen gene expression, and DOX treatment did not alter it significantly in either group. There were no significant differences between untreated HanSD rats and untreated TGR in renin gene expression, and DOX treatment significantly increased it, both in HanSD and TGR (Figure 4B). As shown in Figure 4C, angiotensin-converting enzyme (*ACE*) kidney mRNA expression showed a pattern similar to that for renin gene expression. There were no significant differences between untreated HanSD rats and TGR, and DOX treatment similarly increased it in either strain. There were no significant differences between untreated HanSD rats and untreated TGR in kidney angiotensin-converting enzyme type 2 (*ACE2*) mRNA expression and DOX treatment did not change these values in either group (Figure 4D). As shown in Figure 4E, there were no significant differences between untreated HanSD rats and untreated TGR in kidney mRNA expression of ANG II type 1 (*AT*_1_) receptor, and DOX treatment caused pronounced and similar decreases in this value, similar in either strain. As shown in Figure 4F, there were no significant differences between untreated HanSD rats and untreated TGR in kidney mRNA expression of ANG II type 2 (*AT*_2_) receptor, and DOX treatment significantly decreased it in HanSD rats, but not in TGR. Mas kidney mRNA expression showed a pattern similar to that of AT_2_ receptor gene expression: there were no significant differences between untreated HanSD rats and untreated TGR, and DOX treatment caused a profound decrease in this value in HanSD rats but not in TGR (Figure 4G).

As shown in Figure 5A, untreated TGR showed significantly higher kidney mRNA prepro-Endothelin-1 expression as compared with untreated HanSD rats. DOX treatment significantly increased kidney prepro-Endothelin-1 gene expression in HanSD rats but did not change it in TGR. Likewise, as shown in Figure 5B, untreated TGR showed significantly higher kidney mRNA endothelin-converting enzyme type 1 (*ECE-1*) expression as compared with untreated HanSD rats, and DOX administration elicited significant increases in kidney ECE-1 gene expression in HanSD rats but did not change it in TGR. Untreated TGR showed significantly higher kidney endothelin type A (ET_A_) receptor mRNA expression as compared with untreated HanSD rats (Figure 5C). DOX treatment elicited a significant rise in kidney ET_A_ receptor gene expression in HanSD rats but, in contrast, significantly decreased it in TGR. As shown in Figure 5D, there were no significant differences in endothelin type B (*ET_B_*) receptor mRNA expression between untreated HanSD rats and untreated TGR kidney and DOX treatment did not alter it in either group. Untreated TGR showed significantly higher kidney mRNA cytochrome P-450 (CYP) subfamily 2C23 (*CYP2C23*) expression as compared with untreated HanSD rats (Figure 5E). DOX treatment did not alter kidney this gene expression in HanSD rats but significantly decreased it in TGR. As shown in Figure 3F, there were no significant differences among experimental groups of HanSD rats and TGR in kidney CYP subfamily 4A1 (*CYP4A1*) mRNA expression.

As shown in Figure 6A, there were no significant differences among experimental groups of HanSD rats and TGR in kidney mRNA expression of adrenergic α1a receptors. Untreated TGR showed significantly higher kidney mRNA adrenergic α1b receptor expression as compared with untreated HanSD rats (Figure 6B). DOX treatment elicited a significant rise in kidney adrenergic α1b receptors gene expression in HanSD rats but did not alter it in TGR.

As shown in Figure 6C–E, there were no significant differences in kidney mRNA expression of α2 adrenergic receptors (α2a, α2b, and α2c subtypes) between untreated HanSD rats and untreated TGR. DOX treatment significantly decreased kidney gene expression of all subtypes of α2 adrenergic receptors in HanSD rats but did not alter them in TGR.

There were no significant differences in kidney mRNA expression of β1 and β2 adrenergic receptors between untreated HanSD rats and untreated TGR (Figure 6F,G). As in the case of α2 receptors, DOX treatment elicited significant decreases in kidney gene expression of β1 and β2 adrenergic receptors in HanSD rats but did not change them in TGR.

## 3. Discussion

The first important set of findings relates to the LV mRNA expression of markers that are known to participate in the development and progression of cardiac remodeling in HFrEF [37,38,39] in order to confirm that subsequently obtained kidney mRNA expressions data are associated with cardiorenal syndrome in chemotherapy-induced HFrEF. We found that two weeks after the last DOX injection normotensive HanSD rats, as well as hypertensive TGR, revealed marked increases in the LV *Nppa* expression, a well-known marker of myocardial stress [37,38,39]. Hypertensive TGR also showed increased α actin, skeletal muscle mRNA expression in the LV, a well-known marker for cardiomyocytes dedifferentiation in cardiovascular diseases [40]. In addition, hypertensive TGR also demonstrated decreased LV mRNA expression of β1 adrenergic receptors after DOX treatment, which is an important finding because the downregulation of β1 adrenergic receptors is a typical marker for HFrEF [37,38]. The expression of markers in the LV responsible for the contractile functions, such as MYH6, SERCA, phosholamban, etc., were not altered by DOX treatment. These mRNA expression data are consistent with the knowledge regarding the molecular mechanisms underlying the development and progression of cardiac remodeling in HFrEF [37,38,39]. In addition, we found that DOX administration increased interleukin-6 LV mRNA expression, which is a typical response to DOX treatment [25,28]. However, the gene expression of markers of LV myocardial fibrosis such as TGF-β, collagen α1 type I, and collagen α1 type III were not increased, which suggests that the process of significant myocardial fibrosis has not yet been initiated.

Collectively, based on these findings related to LV mRNA expression, we can confirm that the model of chemotherapy-induced HFrEF by DOX administration reveals typical characteristics of molecular mechanisms activation for HFrEF [37,38,39], suggesting that kidney mRNA data should also be representative.

The second and the most important set of findings of the present study relates to the kidney mRNA expression of selected markers of neurohormonal systems because even if the term cardiorenal syndrome is a simplification as it encompasses a wide spectrum of disorders involving the heart and kidney [7,36], there is no doubt that the interaction between the heart and kidney is crucial in the pathophysiology of the progression of HFrEF [5,6,11,12,41]. Moreover, the compensatory activation of neurohormonal systems in response to the initial insult in HFrEF is first beneficial as it helps maintain stable hemodynamics, but in the long-term, it critically contributes to the progression of HFrEF. Such long-lasting neurohormonal activation, particularly in the kidney, is now considered extremely deleterious, and, therefore, HF remains a life-threatening neurohormonal disorder [5,7,8,42,43,44], and to our best knowledge, it has not been studied in the model of chemotherapy-induced HFrEF yet.

### 3.1. RAAS System

Two weeks after the last DOX injection, normotensive HanSD rats as well as hypertensive TGR, showed marked increases in kidney renin and *ACE* gene expressions and a decrease in *AT*_1_ receptor gene expression. These findings are in good agreement with our recent results showing that in HanSD rats and in TGR, intrarenal ANG II concentrations are markedly elevated after DOX treatment [35]. This supports the notion that in the kidney of chemotherapy-induced HFrEF animals, the vasoconstrictor axis of the RAAS is markedly activated, both at the renin and ACE levels [45,46]. The evidence on inappropriate activation of the main (vasoconstrictor) RAAS axis is supported by the decreased expression of *AT*_1_ receptor gene: consistent with the physiological negative feedback effect of elevated ANG II levels on *AT*_1_ receptor expression [45,46]. On the other hand, DOX treatment did not change the kidney gene expression of *ACE2*, the gene coding critically important enzyme of the vasodilatory axis of the RAAS [47], both in HanSD rats and TGR. In addition, the DOX treatment resulted in substantial decreases in kidney gene expression of the *AT*_2_ and *Mas* receptors genes in HanSD rats and did not change their expressions in TGR. Since activation of the *AT*_2_ and *Mas* receptors genes is underlying the counter-regulatory vasodilator axis of the RAAS [47], our findings indicate that DOX treatment did not activate this axis in TGR and even suppressed it in HanSD rats. This might seem odd in view of our recent findings showing that intrarenal ANG 1-7 concentrations, which is thought to be the most important peptide of the counter-regulatory axis of the RAAS [47], were markedly elevated in HanSD rats as well as in TGR after DOX administration [35]. However, emerging evidence shows a more complex interaction between the vasoconstrictor and vasodilator axes of the RAAS in that ANG 1-7 can antagonize ANG II by mechanisms other than those mediated by the Mas receptors [47]. Thus, the first tentative conclusion is that two weeks after termination of DOX treatment, HanSD rats as well as TGR, show marked intrarenal activation of the vasoconstrictor axis of the RAAS at the mRNA level. RAAS counter-regulatory axis is not appropriately upregulated or is even inappropriately downregulated (again, examined at the mRNA level).

### 3.2. ET System

We showed that untreated TGR have markedly higher kidney prepro-Edothelin-1, *ECE-1*, and *ET_A_* receptor gene expression as compared with untreated HanSD rats, indicating that the vasoconstrictor/sodium retaining axis of the ET system is intrarenally activated in TGR as compared with HanSD rats. These findings at the mRNA level are in agreement with our previous biochemical findings showing that heterozygous, hypertensive TGR (unlike normotensive HanSD rats) have higher kidney concentrations of endothelin-1 (ET-1) [48,49,50]. These findings support the notion that the inappropriately activated ET system contributes to the pathophysiology of hypertension and particularly to the development of hypertension-associated end-organ damage in TGR [49,50,51]. In addition, our results show that two weeks after the last DOX injection, normotensive HanSD rats showed marked activation of the kidney ET system at the mRNA levels, as seen from substantial increases in prepro-Endothelin-1, *ECE-1* and *ET_A_* receptor gene expression. This accords well with the reports that the renal ET system in HF is upregulated and is considered a long-term maladaptive response [48,52]. Remarkably, DOX treatment did not activate the kidney ET system in TGR, and even suppressed *ET_A_* receptor expression. The discrepant response in HanSD rats and TGR is unclear and supports the notion that the pathophysiology of chemotherapy-induced HFrEF might be different in normotensive and hypertensive subjects, particularly when hypertension is accompanied by the initial inappropriate activation of the RAAS.

Hence, the second tentative conclusion is that two weeks after termination of DOX treatment, HanSD rats show considerable intrarenal activation of the ET system at the mRNA level, but this does not occur in TGR.

### 3.3. CYP-Derived Metabolites of Arachidonic Acid (AA)

Our results showed that untreated TGR have higher kidney *CYP2C23* gene expression than untreated HanSD rats, and DOX treatment decreased it in TGR but not in HanSD rats. CYP2C23 is the main enzyme responsible for the intrarenal formation of epoxyeicosatrienoic acids (EETs) via CYP-dependent epoxygenase pathway of AA metabolism [53,54]. EETs exhibit direct vasodilatory effects and also inhibit the renal tubular transport of sodium. It has been proposed that in the kidney, they operate as a protective system counteracting increased intrarenal RAAS activity [55]. The present finding that TGR has increased kidney *CYP2C23* gene expression is in agreement with our previous findings that kidney CYP2C23 protein expression in TGR was higher than in HanSD rats [56,57]. Taken together, the data suggest that under control conditions, the renal activity of EETs in TGR is enhanced or normal and that the DOX-induced intrarenal deficit is likely the consequence of the increased conversion of EETs to biologically almost inactive dihydroxyeicosatrienoic acids (DHETEs) by the soluble epoxide, an enzyme responsible for the fast conversion of EETs to DHETES [53,54,55,56,57]. In addition, in our previous study employing the model of ACF-induced HF, we found that ACF TGR has normal kidney protein expression of CYP2C23 and that the deficit of biologically active EETs compared with healthy TGR was, again, not the result of decreased formation but rather of faster degradation. In contrast, our present findings suggest that in TGR with DOX-induced HFrEF we had to do with decreased kidney EETs formation, as indicated by the profound fall in renal *CYP2C23* gene expression. Kidney CYP4A1 is the main enzyme responsible for the formation of hydroxyeicosatrienoic acids [mainly 20-hydroxyeicosatrienoic acid (20-HETE)] via CYP-dependent ω-hydroxylase pathway of AA metabolism. 20-HETE is thought to be involved in the pathophysiology of hypertension-associated end-organ damage, pathological cardiac hypertrophy, and particularly in the progression of HF [58,59]. Our present results and previous findings in ACF TGR [56] do not support this view and suggest that alterations in CYP-dependent ω-hydroxylase pathway of AA metabolism do not importantly contribute to the volume-overload high-output HF or to the chemotherapy-induced HFrEF after DOX administration.

Thus, the fourth tentative conclusion is that two weeks after termination of DOX treatment HanSD rats do not show any alterations in CYP-dependent epoxygenase or CYP-dependent ω-hydroxylase pathway of AA metabolism. In contrast, our data suggest that DOX treatment caused suppression of CYP-dependent epoxygenase pathway of AA metabolism in TGR.

### 3.4. Adrenergic System

We showed that untreated TGR had substantially higher kidney α1b adrenergic receptor gene expression as compared with untreated HanSD rats. Since activation of these receptors stimulates renal tubular sodium reabsorption [60,61], increased renal sympathetic nervous system (RSNA) could contribute to the pathophysiology of hypertension in TGR, in addition to the role of RAAS hyperactivity. This accords well with our newest findings showing that renal denervation (ablation of both afferent and efferent renal nerves) significantly reduced blood pressure in TGR but not in HanSD rats [12], even though the renal norepinephrine (NE) levels in TGR were not elevated. However, of special interest are our findings after DOX treatment, particularly those in HanSD rats. On the one hand, DOX treatment markedly increased kidney α1b adrenergic receptors gene expression in HanSD rats as compared with their untreated counterparts. This suggests an RSNA-mediated increase in tubular sodium reabsorption in HanSD rats, even though in our recent study we found that intrarenal NE levels were not elevated [35]. On the other hand, DOX treatment in HanSD rats resulted in profound decreases in kidney α2 adrenergic receptor gene expressions (all subtypes). Notably, activation of these receptors might trigger renal tubular cell death, renal inflammation, and initiate renal fibrosis, ultimately leading to chronic kidney diseases [62]. Therefore, downregulation of kidney α2 receptors should be viewed as an appropriate compensatory response. Likewise, DOX treatment suppressed renal β1 adrenergic receptor gene expression in HanSD rats. Since their activation is known to be responsible for increased renin secretion by juxtaglomerular granular cells [45,46,60], such a response should be viewed as appropriate and oppose the action of the increased intrarenal ANG II levels that were reported at this stage in our previous study [35]. Of interest is our finding that DOX treatment distinctly decreased kidney β2 adrenergic receptor gene expression in HanSD rats. However, we cannot propose here potential physiological and/or pathophysiological implications because despite decades of research, their role in the regulation of renal function has not been fully elucidated. It has been suggested that β2 adrenergic receptors also play a role in the regulation of renin secretion, that presynaptic β2 receptors may facilitate NE release, and that they are also involved in the modulation of renal erythropoietin production. However, such respective roles have not been unequivocally defined [60,61,62,63]. Considering the complex interplay among renal adrenergic receptors in the control of intrarenal vessel tone, glomerular and tubular function and/or secretion of hormones, it is critically important to evaluate the role of all adrenergic receptor types. With similar responses in all types and subtypes of adrenergic receptors, one could make some general assumptions regarding the status of kidney adrenergic system under specific situation. In contrast to HanSD rats, DOX treatment did not alter kidney gene expression of any renal adrenergic receptors types in TGR.

Overall, even though two weeks after termination of DOX treatment, HanSD rats showed suppression of kidney gene expression of α2 and β adrenergic receptors, the maintained expression of α1a and particularly increased expression of α1b and with elevated circulating and normal intrarenal NE concentrations [35] suggest that RSNA is higher than in untreated HanSD rats. The maintained kidney gene expression of all adrenergic receptors also indicates that after DOX treatment of TGR, RSNA is inappropriately high.

### 3.5. Potential Clinical Implication and Limitations of the Study

It is important to recognize that direct translation of the present experimental results to clinical practice is not yet feasible, and our study reveals several limitations.

The first set of limitations is generally related to all in vivo experimental models of cancer therapy-associated cardiovascular toxicity as precisely described in the newest scientific statement from the American Heart Association [32]. Most studies are performed in healthy animals, whereas cancer therapy-associated cardiovascular toxicity and subsequent development of cardiomyopathy and finally chemotherapy-induced HF is a tremendously complex process that can be worsened by comorbidities that are very common in patients with cancer diseases. This limitation is also valid for our present study, even if one comorbidity (hypertension) is incorporated in our experimental framework. In addition, it is also important to recognize that anthracycline cardiotoxicity can be augmented by the contemporary use of other anticancer therapies, such as traditional chemotherapy, e.g., cyclophosphamide and/or radiotherapy. In addition, it is also important to acknowledge that not only comorbidities themselves but also pharmacotherapy, which is used for the treatment of the comorbidities and their interactions with anthracycline may play a role in the development of chemotherapy-induced HFrEF. Moreover, on the one hand, it has been shown that cancer cell metabolites promote the development of dilated cardiomyopathy and cardiac dysfunction [64], and on the other hand, that presence of HF is associated with enhanced tumor growth [65]. An appropriate model that would address the complexity of the interplay between all these factors is and probably will be an unmet need for a long time, but it is important that at least some aspects, e.g., cardiovascular comorbidities, should be considered and included in preclinical models of chemotherapy-induced HFrEF and our recent [35] and present study represent such efforts.

The second set of limitations relates to the fact that the analysis was performed solely at the mRNA level. We are fully aware that the complexity of various interplays between neurohormonal systems requires more comprehensive analysis (i.e., gene and protein expression as well as radioligand studies in case of receptor analysis), and, therefore, the solitary analysis might sometimes lead to some misleading assumptions. We fully agree with the notion formulated by Giebish [66] that current nephrology should encompass an interdisciplinary approach, not only focus on cellular aspects, without evaluating their physiological relevance on the level of the whole organ and organism. Thus, our study should be considered only as of the initial stage for further research. However, we are convinced that the presented findings and conclusions constitute a valuable and solid basis for such studies in the near future. In addition, there has also been a growing recognition that in cancer therapy-related cardiac dysfunction, the development of endothelial dysfunction plays an important role, and it is particularly true for chemotherapy-induced cardiac damage elicited by anthracycline treatment [31], but this issue has not been evaluated in our present study and it is additional shortcoming of our current study and again future studies should also address this issue.

The third set of limitations relates to the lack of a more in-depth evaluation of the underlying mechanism(s) responsible for the cardio-renal toxicity of DOX treatment. However, it should be acknowledged that mechanisms of anthracyclines-induced cardiotoxicity are already well-recognized, including direct cardiomyocyte cytotoxicity via topoisomerase II-mediated DNA damage, generation of reactive oxygen species (ROS), and subsequently impaired mitochondrial function [67,68]. The mechanism(s) underlying anthracycline (particularly doxorubicin)-induced cardiotoxicity were recently reviewed [25,28]. Nevertheless, it is important to highlight that our present study has not been focused on evaluating mechanisms and/or determinants of DOX-induced cardio-nephro toxicity but on the characterization of kidney responses at the mRNA level in the initial phase of chemotherapy-induced HFrEF.

### 3.6. Conclusions

**Figure 7:**
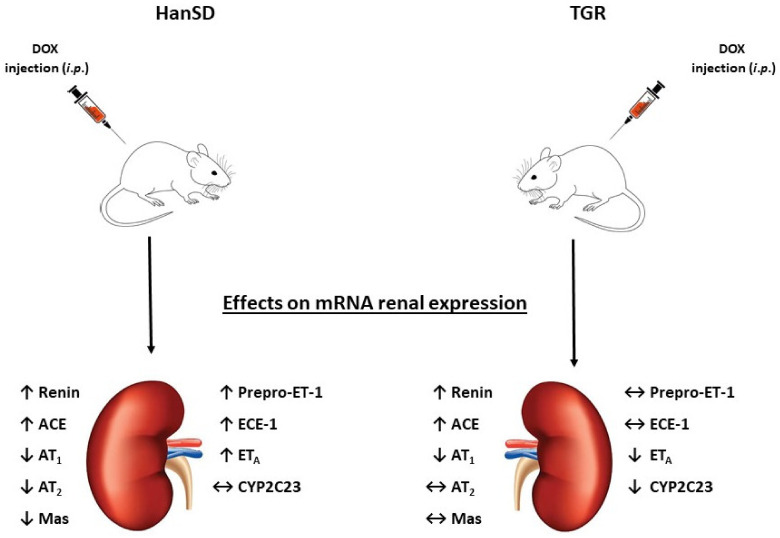
Graphical summary of the most important effects of doxorubicin (DOX) treatment on the intrarenal messenger ribonucleic acid (mRNA) expressions of vasoactive systems implicated in the pathophysiology of heart failure-associated cardiorenal syndrome in normotensive, transgene-negative Hannover Sprague–Dawley (HanSD) rats and hypertensive, *Ren-2* transgenic (TGR) rats. Specifically, renin, angiotensin-converting enzyme (ACE), angiotensin II type 1 (AT_1_) receptor, angiotensin II type 2 (AT_2_) receptor, Mas receptor (Mas), prepro-endothelin-1 (Prepro-ET-1), endothelin-converting enzyme type 1 (ECE-1), endothelin type A (ET_A_) receptor and cytochrome P-450 subfamily 2C23 (CYP2C23). (↓) indicates downregulation, (↑) indicates upregulation, (↔) indicates no significant change.

In general, our findings support the notion that DOX-induced HFrEF is a suitable model to study pathophysiological aspects of this disorder. The most important findings at the initial phase of DOX-induced HFrEF are graphically summarized in Figure 7 and recapitulated as follow:

First, both strains display pertinent mRNA changes indicating marked intrarenal activation of the classical axis of the RAAS combined with either absolute or relative deficiency of its counter-regulatory axis.

Second, HanSD rats but not TGR display substantial activation of the ET system.

Third, it appears that TGR but not HanSD rats show a deficiency of the intrarenal CYP-dependent epoxygenase pathway of AA metabolism.

These results constitute a solid basis for future studies evaluating the pathophysiology of cardiorenal syndrome secondary to chemotherapy-induced HFrEF in order to develop novel treatment strategies for chemotherapy-induced HFrEF.

## 4. Methods

### 4.1. Ethical Approval, Animals and HF Induction

All animals used in the present study were bred at the Center for Experimental Medicine of this Institute from stock animals supplied by the Max Delbrück Center for Molecular Medicine, Berlin, Germany. Heterozygous TGR were generated by breeding male homozygous TGR with female homozygous HanSD rats as described in the original study [69], age-matched HanSD rats served as transgene-negative normotensive controls. The animals were kept on a 12-h/12-h light/dark cycle. Throughout the experiments, rats were fed a normal salt, normal protein diet (0.45% NaCl, 19–21% protein) produced by SEMED (Prague, Czech Republic) and had free access to tap water. Male TGR and HanSD rats, at the initial age of 9 weeks, derived from several litters, were randomly assigned to the experimental groups. DOX-induced HFrEF was obtained by methods that was originally developed more than 30 years ago [27,34] and recently characterized in our laboratory [35]. The procedure consisted of 6 intraperitoneal (*i.p.*) injections of DOX (2.5 mg/kg of body weight) given over 2 weeks; the cumulative dose was 15 mg/kg of body weight, and throughout this period, no mortality was observed. This sample reflects well the human clinical circumstances; the cumulated dose corresponded to 550–600 mg/m^2^ body surface applied in patients. The incidence of DOX-induced cardiomyopathy with this dose was usually 26%, and even higher in hypertensive patients [22,23,24,25]. Control animals received vehicle solution in the same volume (saline solution with lactose at the same concentration as used for dilution of DOX).

### 4.2. Detailed Experimental Design

#### 4.2.1. Assessment of the Effects of DOX on Kidney mRNA Expression

Two weeks after the last injections, the animals were killed by an overdose of thiopental sodium given *i.p.* (about 250 to 300 mg) and organs were weighed. Kidney tissue samples were immediately harvested into liquid nitrogen and stored at −80 °C until analysis. The following experimental groups of animals were examined (*n* = 9 in each group):

1. HanSD rats + vehicle
2. TGR + vehicle
3. HanSD rats + DOX
4. TGR + DOX

The tissue mRNA expression was determined by the standard technique described in our previous studies [70,71]. The measurement of multiple mRNA expression was performed in accordance with the manufacturer’s instructions (384-well microfluidics TaqMan array cards; custom setting of selected genes; Applied Biosystems, Foster City, CA, USA). The genes, which were investigated are listed below, including the appropriate ID assay identification number and abbreviation given by the manufacturer (Table 1 and Table 2). In the heart tissue well-known markers of myocardial stress, inflammation, and myocardial fibrosis were chosen to be evaluated [37,38,39]. 

The selection strategy was based on our requirement to perform an immensely comprehensive analysis. Therefore, we wanted to include all potential pathways of the particular neurohormonal system. In the case of RAS, the gene for the main precursor for both pathways (angiotensinogen) were analyzed, and all the genes of enzymes responsible for the production of biologically active products of both pathways (i.e., for production of ANG II and ANG 1-7) and finally genes of receptors responsible for biological actions of ANG II and ANG 1-7 (i.e., AT_1_, AT_2_, and Mas receptor).

#### 4.2.2. Relative Gene Expression Calculation

In all experiments, relative gene expression was calculated by the *2^−ΔΔCt^* method, which was the most frequently used for such experiments [70,72]. This method directly used the *Ct* (*threshold cycle*) information generated from a qPCR system. Ct was the cycle number where the fluorescence generated by the PCR produce was distinguishable from the background noise. To calculate relative gene expression in target and reference samples, a housekeeping gene was used as the normalizer. 18S rRNA was used as the normalizer because its expression level remained relatively stable in response to any treatment [72,73] and it meets the “Minimum Information for Publication of qPCR Experiments” guidelines as introduced by Bustin et al. [74].

Firstly, Δ*Ct* of each sample was calculated:

Δ*Ct* = *Ct* (gene of interest) − *Ct* (housekeeping gene)

The expression of mRNA of selected genes was related to a control group, i.e., HanSD rats treated with vehicle. The final results were expressed as the *n*-fold difference in gene expression of mRNA of target genes between the appropriate experimental group and control group calculated as follows:

*n*-fold expression = 2^−(Δ*Ct* of experimental group − Δ*Ct* of control group)^ (1)

Subsequently, the log transformation of the data was performed to make it more symmetrical as recommended and generally accepted for the evaluation of relative gene expression results [72,74,75]. Thus, the values in the graphs represent log2 *n*-fold gene expression.

### 4.3. Statement of Ethics

The study followed the guidelines and practices established by the Animal Care and Use Committee of the IKEM, which accorded with the national law and were approved by the Animal Care and Use Committee of the IKEM and, consequently, by the Ministry of Health of the Czech Republic (project identification code 36388/2019-4/) 15 August 2019.

### 4.4. Statistical Analysis

Statistical analyses were performed using Graph-Pad Prism software v7.0 (Graph Pad Software, San Diego, CA, USA). Statistical comparison was made by one-way ANOVA when appropriate, and all data were also analyzed by D’Agostino-Pearson normality test, which was currently recommended as the best approach to quantify how far the distribution is from Gaussian in terms of symmetry and shape, particularly for data obtained by qRT-PCR [75]. Values are expressed as mean ± SEM. The values of *p* below 0.05 were considered statistically significant.

## Figures and Tables

**Table 1:** The genes analyzed in the LV tissue.

ID Assay	Gene Name	Abbreviation
Rn00664637_g1	natriuretic peptid A	*Nppa*
Rn01488781_g1	myosin, heavy chain 6, cardiac muscle, alpha	*Myh6*
Rn01488777_g1	myosin, heavy chain 7, cardiac muscle, beta	*Myh7*
Rn01426628_g1	actin, alpha 1, skeletal muscle	*Acta1*
Rn00568762_m1	ATPase, Ca++ transporting, cardiac muscle, slow twitch 2	*SERCA*
Rn01463848_m1	collagen, type I, alpha 1	*Col1a1*
Rn01437681_m1	collagen, type III, alpha 1	*Col3a1*
Rn00824536_s1	adrenoceptor beta 1	*Adrb1*
Hs99999901_s1	18S rRNA ribosomal subunit	*18s rRNA*
Rn00572010_m1	transforming growth factor, beta 1	*Tgfb1*
Rn01434045_m1	phospholamban	*Pln*
Rn00572711_m1	Interleukin-6	*IL-6*

**Table 2:** The genes analyzed in the kidney cortex.

ID Assay Number	Gene Name	Abbreviation
Rn00561847_m1	renin	*Ren*
Rn00561094_m1	angiotensin I converting enzyme	*Ace*
Rn01416293_m1	angiotensin I converting enzyme 2	*Ace2*
Rn00593114_m1	angiotensinogen (serpin peptidase inhibitor, clade A, member 8)	*Agt*
Rn02758772_s1	angiotensin II receptor, type 1a	*Agtr1a*
Rn00562673_s1	MAS1 proto-oncogene, G protein-coupled receptor	*Mas1*
Rn00561129_m1	prepro-Endothelin 1	*Edn1*
Rn00585943_m1	endothelin converting enzyme 1	*Ece1*
Rn00561137_m1	endothelin receptor type A	*Ednra*
Rn00569139_m1	endothelin receptor type B	*Ednrb*
Hs99999901_s1	18S rRNA ribosomal subunit	*18s rRNA*
Rn00598510_m1	cytochrome P450, family 4, subfamily a, polypeptide 1	*Cyp4a1*
Rn01413752_m1	cytochrome P450, family 2, subfamily c, polypeptide 23	*Cyp2c23*
Rn00567876_m1	adrenoceptor alpha 1A	*Adra1a*
Rn01471343_m1	adrenoceptor alpha 1B	*Adra1b*
Rn00562488_s1	adrenoceptor alpha 2A	*Adra2a*
Rn00593312_s1	adrenoceptor alpha 2B	*Adra2b*
Rn00593341_s1	adrenoceptor alpha 2C	*Adra2c*
Rn00824536_s1	adrenoceptor beta 1	*Adrb1*
Rn00560650_s1	adrenoceptor beta 2, surface	*Adrb2*
Rn00560677_s1	angiotensin II receptor, type 2	*Agtr2*

## Data Availability

Not applicable.

## Abbreviations

AA	arachidonic acid
ACEi	angiotensin converting enzyme inhibitor
ACE	angiotensin converting enzyme
ACE2	angiotensin converting enzyme type 2
ACF	aorto-caval fistula
ANG II	angiotensin II
ANG 1-7	angiotensin 1-7
AT_1_	angiotensin II type 1 receptor
AT_2_	angiotensin II type 2 receptor
CYP	cytochrome P-450 enzyme
CYP2C23	cytochrome P-450 enzyme subfamily 2C23
CYP4A1	cytochrome P-450 enzyme subfamily 4A1
DHETEs	dihydroxyeicosatrienoic acids (DHETEs)
DOX	doxorubicin
ECE-1	endothelin-converting enzyme type 1
EETs	epoxyeicosatrienoic acids
ET_A_	endothelin type A receptor
ET_B_	endothelin type B receptor
ET-1	endothelin-1
HanSD	normotensive, transgene-negative, Hannover Sprague–Dawley rats
HF	heart failure
HFrEF	heart failure with reduced ejection fraction
IKEM	Institute for Clinical and Experimental Medicine
MI	myocardial infarction
mRNA	messenger ribonucleic acid
NE	norepinephrine
RAAS	renin-angiotensin-aldosterone system
ROS	reactive oxygen species
RSNA	renal sympathetic nerve activity
sEH	soluble epoxide hydrolase
TGR	Ren2 renin transgenic, hypertensive rats
20-HETE	20-hydroxyeicosatrienoic acid
